# A biological agent modulates the physiology of barley infected with *Drechslera teres*

**DOI:** 10.1038/s41598-021-87853-0

**Published:** 2021-04-15

**Authors:** Aurélie Backes, Nathalie Vaillant-Gaveau, Qassim Esmaeel, Essaid Ait Barka, Cédric Jacquard

**Affiliations:** Université de Reims Champagne-Ardenne, Unité de Recherche Résistance Induite et Bio-protection des Plantes, RIBP - EA 4707 - USC INRAE 1488, Moulin de la Housse-Bâtiment 18, BP 1039, 51687 Reims Cedex 2, France

**Keywords:** Non-photochemical quenching, Photosystem I, Photosystem II, C3 photosynthesis, Fungal host response, Plant physiology, Biotic

## Abstract

Recognized as the causal agent of net blotch, *Drechslera teres* is responsible for major losses of barley crop yield. The consequences of this leaf disease are due to the impact of the infection on the photosynthetic performance of barley leaves. To limit the symptoms of this ascomycete, the use of beneficial bacteria known as “Plant Growth Promoting Rhizobacteria” constitutes an innovative and environmentally friendly strategy. A bacterium named as strain B25 belonging to the genus *Burkholderia* showed a strong antifungal activity against *D. teres*. The bacterium was able to limit the development of the fungus by 95% in detached leaves of bacterized plants compared to the non-bacterized control. In this study, in-depth analyses of the photosynthetic performance of young barley leaves infected with *D. teres* and/or in the presence of the strain B25 were carried out both in and close to the necrotic area. In addition, gas exchange measurements were performed only near the necrotic area. Our results showed that the presence of the beneficial bacterium reduced the negative impact of the fungus on the photosynthetic performance and modified only the net carbon assimilation rate close to the necrotic area. Indeed, the presence of the strain B25 decreased the quantum yield of regulated non-photochemical energy loss in PSII noted as Y(NPQ) and allowed to maintain the values stable of maximum quantum yield of PSII photochemistry known as F_v_/F_m_ and close to those of the control in the presence of *D. teres*. To the best of our knowledge, these data constitute the first study focusing on the impact of net blotch fungus and a beneficial bacterium on photosynthesis and respiratory parameters in barley leaves.

## Introduction

The hemibiotrophic ascomycete *Drechslera teres* is the pathogen responsible for net blotch on barley^1,2^. This foliar disease is considered as the most important threatening disease since it affects barley crop production worldwide, mainly in regions with long wet weather periods and temperature between 15 and 20 °C^3–5^. Net blotch symptoms include the expansion of reticulated brown stripes accompanied by necrosis and chlorosis of barley leaves. These first symptoms appear as early as 48 h after inoculation of the pathogen. Nowadays, several means exist to control the disease, mainly via fungicides application^6–9^. The environmental laws, the proliferation of initiatives for amore eco-friendly agriculture, as well as the appearance of resistance of the fungus to fungicides, have been contributed to the development of new alternatives including the use of beneficial bacteria known as “Plant Growth Promoting Rhizobacteria” (PGPR)^10–15^.

PGPR present abundantly in the rhizosphere have beneficial effects on plants. These microorganisms colonize plant roots, increase stem emergence and stimulate plant growth through several mechanisms. In addition, PGPR can facilitate plant development by improving the availability of certain nutrients, by producing hormones, or by limiting the pathogen growth via direct or indirect mechanisms^16–19^. Some of these organisms are also able to induce resistance in the plant subjected to biotic or abiotic stress. Bacteria belonging to the genus *Burkholderia* represent a group of 90 species isolated from several ecological niches. Within recent years there has been great interest in *Burkholderia* regarding biotechnology and, more particularly, in the biocontrol of plant diseases^20,21^. Recently, it has been proposed to divide the genus *Burkholderia* into seven distinct groups; these include *Burkholderia* sensu stricto, *Trinickia*, *Caballeronia*, *Robbsia*, *Pararobbsia*, Mycetohabitans, and *Paraburkholderia.* The latter includes mainly environmental species mostly reported to be associated with plants and have biocontrol and bioremediation properties^22,23^. In the present study, a strain of *Burkholderia*, referred to as strain B25, isolated from maize rhizosphere cultivated in France was selected for its antagonistic activity against net blotch fungus. Its antagonistic activity against *D. teres* was demonstrated by using the dual culture method on PDA plates as well as on detached barley leaves assay.

The yield losses induced by *D. teres* infection can be explained by a reduction in photosynthesis^24,25^. The major effects caused by the pathogens on the photosynthetic mechanisms of plants include impairment in energy dissipation by chlorophyll (Chl) *a* fluorescence, reduction in gas exchange rates, increase in foliar temperature and limitation in mesophyll conductance^26^. Chl *a* fluorescence imaging and measurement combined with gas exchange measurements are key indicators of in situ photosynthetic performance of plants. Measuring Chl *a* fluorescence is nondestructive, non-invasive and a sensitive technique providing information on the physiological state of infected plants. The variable-to-maximum Chl *a* fluorescence ratio, also called maximal quantum yield of dark-adapted leaves (F_v_/F_m_) is close to 0.8 in healthy leaves^27^. F_v_/F_m_ represents also the maximum quantum yield of photosystem II (PSII) photochemistry. To compare non-infected with host tissue infected by the pathogen, the parameter Fv/Fm is one of the most important parameters^28^. The energy absorbed by PSII can be lost in the photochemical form Y(II) or in the non-photochemical form. The non-photochemically lost energy is itself divided into two pathways known as the yield induced by regulated non-photochemical energy loss (Y(NPQ)) and the yield for other energy losses (Y(NO)). Furthermore, the presence of chlorotic and necrotic areas lead to a decrease in the photosynthetic production of assimilates. The negative effects of pathogens on photosynthetic parameters in different plants have been described in many reports^29–33^.

During several interactions between host–pathogen such as *Pseudomonas syringae* and *Arabidopsis thaliana*^34^, *Puccinia recondita* or *Blumeria graminis* and wheat^35^, *Colletotrichum lundemuthianum* and bean^36^, *Monographella albescens* and rice^37^, *Rhynchosporium secalis* and barley^38^, *Puccinia hordei* and barley^39^, *Puccinia psidii* and *Eucalyptus urophylla*^40^, *Bipolaris oryzae* and rice^41^, the measurements indicate a net decrease of CO_2_ assimilation, stomatal conductance, photosynthetic electron transport rate and transpiration ratio, accompanied by a loss of photosynthetic yield.

Conversely, it has been shown that biocontrol agents can counteract the pathogens effects by enhancing photosynthetic activity. For instance, *Streptomyces thermocarboxydus* used as biocontrol agent allows to control *Fusarium* and enhance yield by increasing photosynthesis in tomato^42^. Moreover, it has been demonstrated that the two plant growth promoting organisms, the fungus *Rhyzophagus irregularis* and the bacterium *Bacillus amyloliquefaciens* enhance the photosynthetic efficiency in addition to the shoot weight increase in *Trifolium repens* and *Fragaria vesca*^43^.

The present study aimed to investigate the modifications on photosynthesis process in barley plantlets during infection with *D. teres* and in the presence of strain B25 used as biocontrol agent. Imaging PAM observations and measurements with *MONITORING-PAM* and GFS 3000 demonstrated the impact of the pathogen causing barley net blotch and strain B25 on the barley. Therefore, these results serve as a basis to demonstrate the potential role of biocontrol agents used in environmentally friendly agriculture and highlight the interest of using beneficial bacteria to protect crops against phytopathogens.

## Results and discussion

### Antifungal activity of strain B25

Strain B25, isolated from maize rhizosphere cultivated in the Marne department, France was screened for its ability to inhibit the growth of *D. teres*. The strain B25 was active against the pathogen by showing a strong antifungal activity with the results obtained on PDA plates (Figure S1). To further evaluate the ability of the strain to inhibit the development of the pathogen at the plant level, the antifungal activity of the strain B25 was investigated on detached leaves of barley. Disease symptoms were evaluated by measuring the necrosis diameter. As shown in Figure S2, the bacterium was able to limit the development of the fungus by 95%.

### Impact of *D. teres* infection on Chl *a* fluorescence in barley

**Figure 1:**
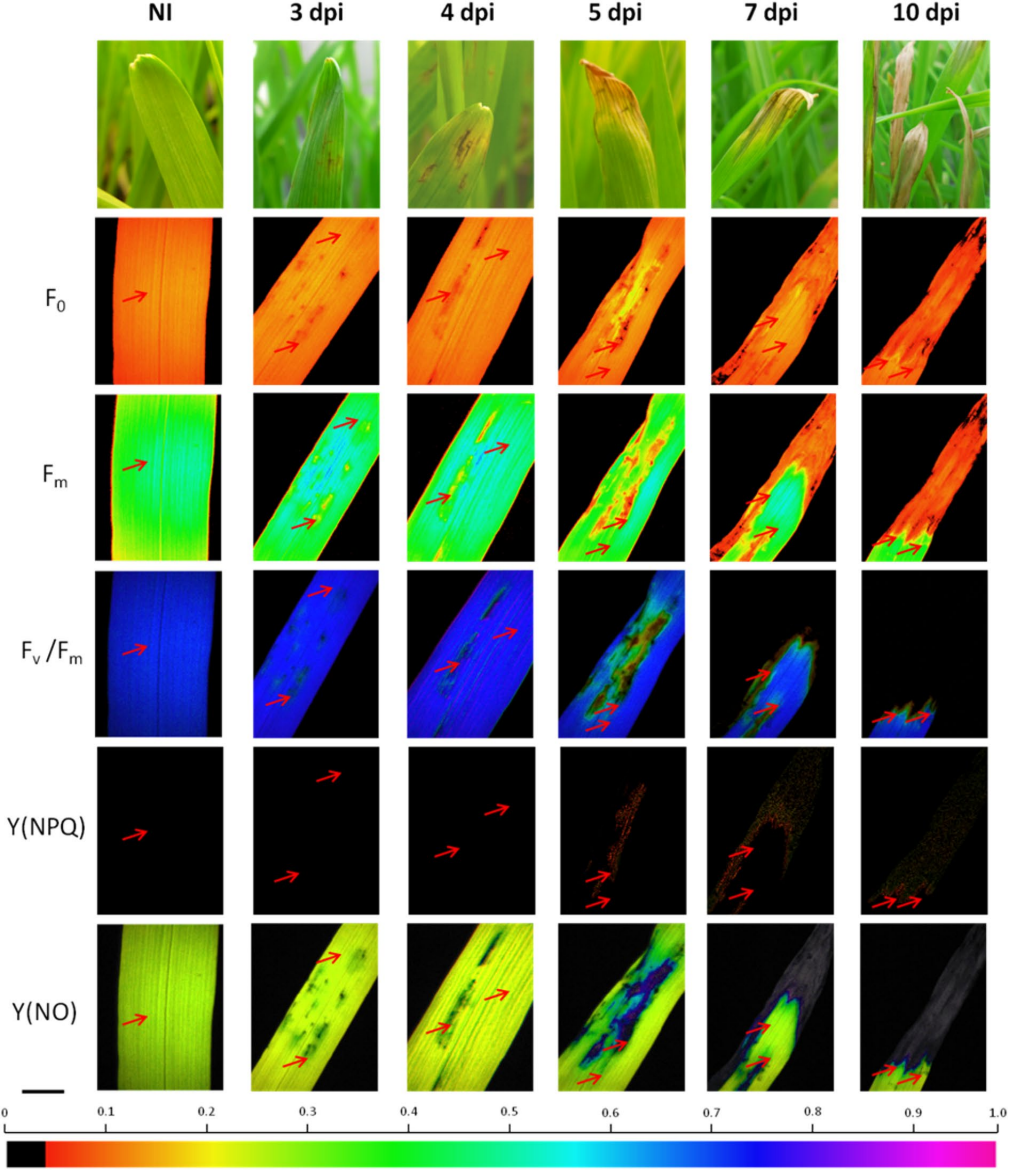
Parameters of chlorophyll *a* fluorescence (F_0_), maximal fluorescence (F_m_), maximal photosystem II quantum yield (F_v_/F_m_), the quantum yield of regulated non-photochemical energy loss Y(NPQ) and quantum yield of non-regulated energy dissipation Y(NO) determined on the leaflets of barley plants non inoculated (NI) or inoculated with *D. teres* at 3, 4, 5, 7 and 10 dpi. The red arrows indicate the measurement zones. Bar 0.5 cm.

During the development of the disease, darker or even black spots appeared. Over time, these points lengthened and swelled to form stripes (Fig. 1). At 3 days after inoculation with *D. teres,* the first visual changes in Chl *a* fluorescence images were present on the leaflets from inoculated barley (Fig. 1). The non-infected plants (NI) showed an orange color for F_0_ parameter. This parameter provides information on the minimal level of fluorescence emission when all of the PSII reactions centers are “closed”. In our case, the symptoms caused by *D. teres* led to a decrease of F_0_ at the infection start (3 dpi), then to an increase (yellow-color) at 5 dpi and finally to a decrease of this parameter at 10 dpi. The parameter variation was localized in the penetration area of the fungus.

In addition to the F_0_ parameter, the maximum chlorophyll fluorescence yield (F_m_) was measured in a dark-acclimated barley leaves. F_m_ parameter is indicated as a green–blue color for the non-infected leaf tissues (NI) with values close to 0.4–0.5. According to Fig. 1, F_m_ parameter is visualized by a green or yellow color at different foliar area at the beginning of the experiment at 3 and 4 dpi. F_m_ parameter was decreased or increased locally from 5dpi.

These variations became more pronounced with the progression of the disease. This change is the result of the massive necrosis extending from the apex to the base of the leaf. In addition, the F_v_/F_m_ parameter was altered at the level of the fungus penetration area at 3 and 4 dpi. This alteration was noticed by the passage from a blue color to a black color indicating the appearance of necrosis on the leaves.

A decrease of F_0_ and F_m_ parameters in infected plants has been reported by Dias et al*.*^28^. More precisely, the presence of *Colletrichum truncatum* on soybean leaves decreased the light efficiency collecting excitation energy^28^. Being a sensitive indicator of photosynthetic performance, the F_v_/F_m_ parameter was close to 0.8 in healthy plants. The results obtained clearly show that the development of *D. teres* symptoms on barley leaves decreases the values of F_v_/F_m_. Similar results were obtained for other plant-pathogen interactions^44,45^.

On the contrary, Y(NPQ) parameter increased during the infection and more particularly, at 5 dpi with *D. teres*. In fact, a transition from black to red-green color was visualized for Y(NPQ) with values changes from 0 to 0.200, respectively. This slight increase in the values of the Y(NPQ) parameter indicates regulation by heat dissipation of the barley leaf to protect against excessive excitation energy^46^. In the presence of the net blotch symptom on barley leaves, a change from green-yellow to blue color appeared for the Y(NO) parameter before having no yield symbolized by the black color. Y(NO) parameter increased in leaf areas attacked by the fungus (Fig. 1). The energy absorbed by the PSII is either used in photochemistry (Y(II)) or non-photochemically lost which can be divided into processes associated with controlling thermal dissipation (Y(NPQ)) and processes associated with other energy losses (Y(NO))^46,47^. High Y(NPQ) values indicate heat dissipation. However, high values of Y(NO) represent an excess excitation energy reaching the reaction centers. This excess energy results in a strong reduction of PSII acceptors, and consequently, in an increase of oxidative stress such as the formation of reactive oxygen species^48^. Therefore, the high Y(NPQ) values are evidence of the capacity of photoprotection for plants submitted to stress^47^.

The appearance of these symptoms cause an alteration of the photosynthetic mechanism, as well as a loss of optical properties in barley leaf which ends up being totally necrotic at the point of penetration of the fungus. For the rest of this study, all of the other photosynthetic or respiratory parameters were measured on barley leaves close to the necrotic area. Indeed, as presented in Fig. 1, the necrotic areas did not show activity while the area close to the necrosis (red arrows) presented major changes in photosynthetic mechanisms.

### Monitoring of Y(II), ETR and PAR parameters in barley leaves

**Figure 2:**
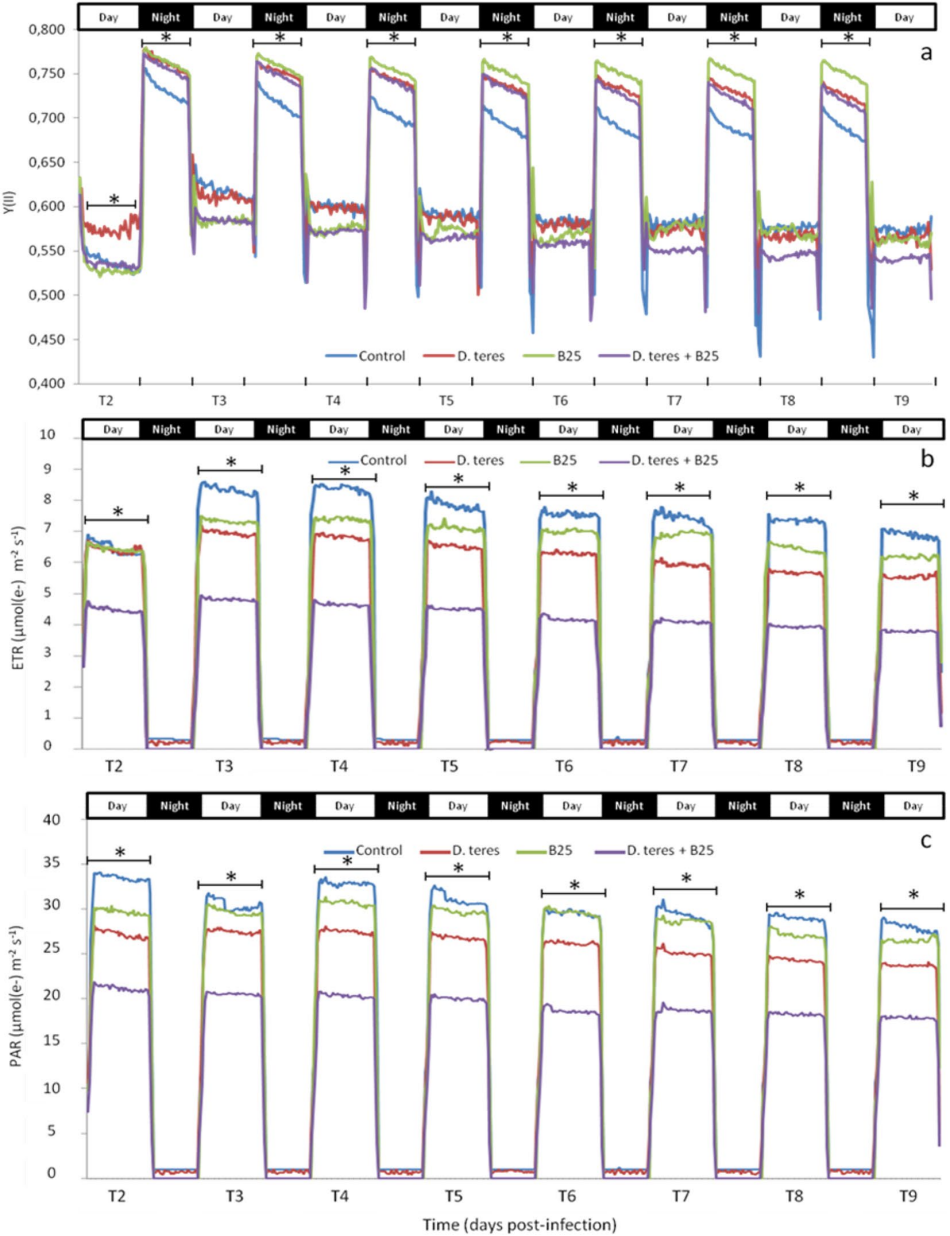
Monitoring of the Y(II) (**a**), ETR (**b**) and PAR (**c**) parameters as a function of time for different experimental conditions: control barley (blue), barley infected with *D. teres* (red), barley bacterized with strain B25 (green) and barley infected and bacterized (purple). The data correspond to the mean of three independent experimental replicates, each with three or four plants per treatments (n = 9 or 12). Data were obtained from 2 to 9 dpi noted in this figure T2 to T9. Measurements were taken every 20 min. Asterisks (*) show the significant differences between the experimental conditions during a period of day or night for a time-point (Student’s test, *p* value < 0.05).

The monitoring of the Y(II), ETR and PAR parameters was carried out continuously thanks to the PAM Monitoring. The data shown in Fig. 2 represent the means of three independent experimental replicates, each was realized with three (control and bacterial barley condition) or four (infected barley and bacterial–infected barley conditions) plants per treatments. During the day, Y(II) measurements reflect the ФPS (II) representing the effective quantum yield of PSII photochemistry and during the night, Y(II) measurements represent the F_v_/F_m_ called the maximum quantum yield of PSII photochemistry.

According to the results obtained, Y(II) decreased during the day to values between 0.550 and 0.600 and increased at the night with values close to 0.750 (Fig. 2a). The data also demonstrated variations of the Y(II) parameter depending on the experimental conditions. In daytime condition, the condition of barley infected only with *D. teres* showed significantly higher ФPS (II) values (0.580) compared to other conditions at 2 dpi (0.540). This result was not found for the other daytime periods of the experiment. At 2 dpi in daytime condition, barley plants were placed under a bell during two days in order to increase the humidity rate and favoring the development of *D. teres* and hence the parameters monitoring began using Monitoring PAM*.* Therefore, 2 dpi represents the time point where *D. teres* was in optimal growth conditions which could explain the obtained result. From 3 to 6 dpi, the ФPS(II) values were lower in the presence of strain B25. As the disease developed, bacterized barley plants with strain B25 and infected with *D. teres* showed the lowest ФPS(II) values from 7 to 9 dpi compared to other conditions, but these values were not significant. This last tendency in daytime condition can be explained by the fact that barley plant can perceive the bacteria and also the pathogen as external agents. The plant host may have closed its stomata to prevent entry of the pathogen. As a consequence, a stomata closure may induce a decrease the effective quantum yield of PSII photochemistry (ФPS(II))^49^.

For the night condition, the same trend in monitoring of parameter F_v_/F_m_ was present from 2 to 8 dpi (Fig. 2a). The F_v_/F_m_ values for the control were significantly different compared to other experimental conditions in the night period from 2 to 8 dpi. Indeed, the control condition showed the lowest values close to 0.700 compared to the other experimental conditions with the values close to 0.750. In addition, barley plants treated only with the strain B25 showed a maintenance of the F_v_/F_m_ values during all period of the experiment. A significant difference between this condition and other conditions tested was noted from 5 dpi.

The electron transport rate (ETR) was calculated using Y(II) and the PAR parameter. Therefore, a decrease in Y(II) might be related to an alteration of the ETR parameter. According to Fig. 2b, ETR has values close to 0 for all the experimental conditions in night conditions. In daytime conditions, significant differences were noted. At 2 dpi, the “*D. teres* + B25” condition showed significantly lower values (Student’s test; *p* value < 0.05) compared to the other experimental conditions. Then, the same profile of ETR means was displayed from 3 to 9 dpi in daytime conditions. More particularly, “*D. teres* + B25” condition had the lowest values and which were similar to 2 dpi. Then, the infected condition noted “*D. teres*” showed significantly lower means compared to B25 and control conditions. The PAR values follow the similar trend as the ETR parameter, both for the night and the day conditions (Fig. 2c). Indeed, the control plants showed significantly higher PAR means compared to the other conditions from 2 to 9 dpi. On the contrary, B25-bacterized plants infected with *D. teres* had significantly lower PAR means by pairwise comparison with the other conditions. This indicates that *D. teres* and the strain B25 clearly affect PAR and ETR parameters.

The presence of strain B25 allowed to maintain the maximum efficiency of the PSII. However, under daytime conditions, the combination of the fungus and the bacterium decreased this efficiency of PSII and was lower than the value measured on barley infected with *D. teres*. In the literature, it is indicated that many PGPR strains, including *Azospirillum brasilence*^50^, *Bacillus substilis* GB03^51^, *B. phytofirmans*^52^ and *Pseudomonas fluorescens*^53^ improve the PSII activity. Grapevine seedlings inoculated with *B. phytofirmans* showed higher photosynthetic activity than non-bacterized seedlings^54^. Studies have shown that strain PsJN induced a cell wall strengthening in *A. thaliana* similar to that observed after cold exposure^55^. Indeed, the cell wall architecture is important in resistance to abiotic or biotic stress. It has been shown that the cell wall involves in interactions between plants and their environment hence its composition and structure change to limit further pathogen spread^56^.

### Variation of PSI and PSII activity regulation in barley under stress

**Figure 3:**
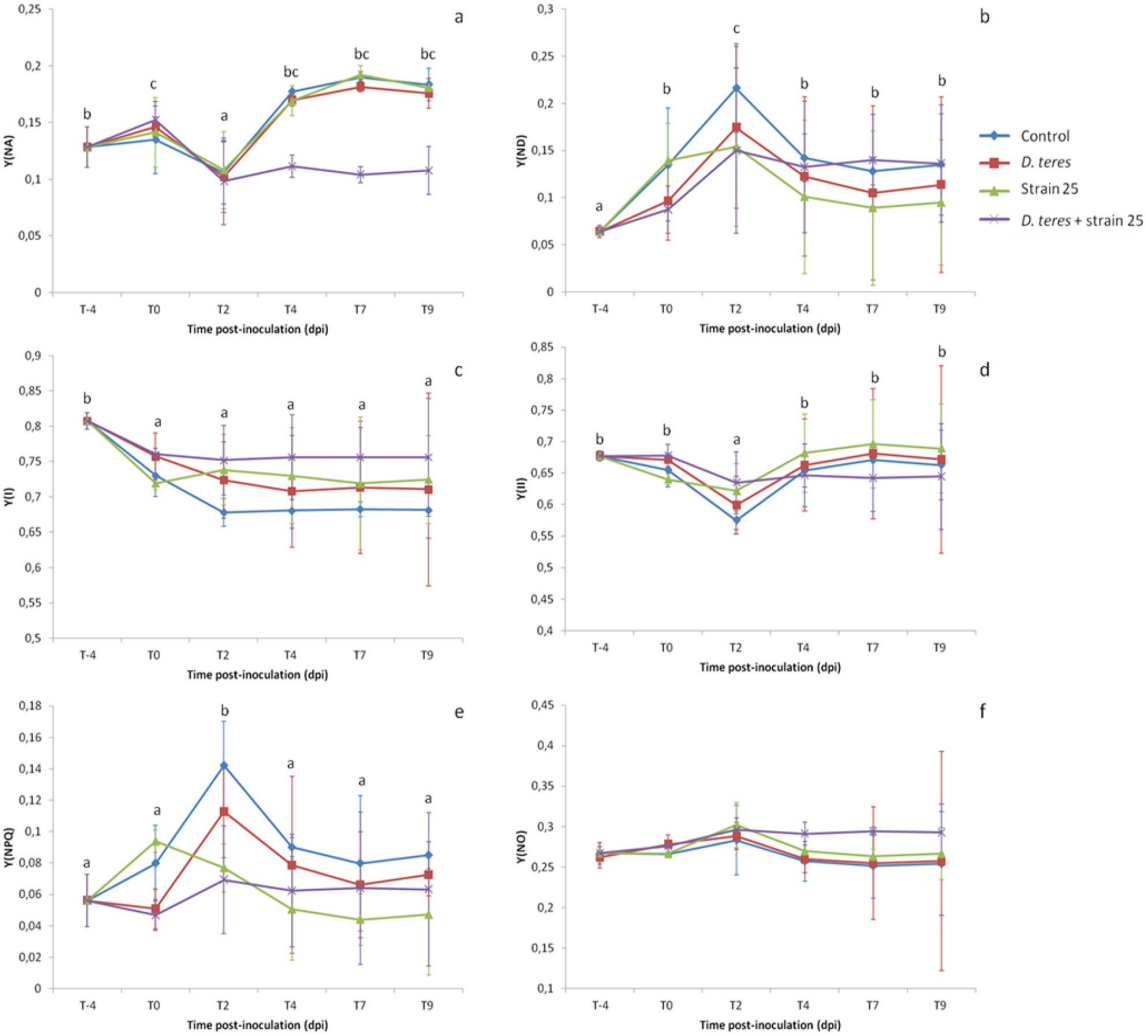
Variations of PSI parameters with PSI acceptor side limitation Y(NA), PSI donor side limitation Y(ND) and efficient quantum yield of PSI Y(I) in barley leaf accompanied by changes of PSII parameters with quantum yield of regulated energy dissipation Y(NPQ), quantum yield of non-regulated energy dissipation Y(NO) and efficient quantum yield of PSII Y(II). The measurements were performed with several experimental conditions: control barley, barley infected with *D. teres*, barley bacterized with strain B25 and barley infected with *D. teres* in combination with strain B25. These data were obtained 4 days before inoculation with *D. teres* (T-4), 0 days before (T0 BP) or after pulverization of the pathogen (T0 AP), 2, 4, 7 and 9 dpi with *D. teres* noted T2, T4, T7 and T9, respectively. The mean was calculated from three independent experiments for each experimental condition and for each time-point, n = 15. Different letters indicate statistically different means (Student’s test; *p* value < 0.05) between times points.

Dual PAM-100 and GFS-3000 measure simultaneously the PSI and PSII parameters. Concerning the PSI parameters, Y(NA) represents the over-reduction of the PSI acceptor side leading to the PSI photoinhibition (Fig. 3a). According to the averages obtained, Y(NA) tended to decrease at 2 dpi for all experimental conditions with significantly different values by comparison two by two with the other times-points (Student’s test; *p* value < 0.05). From 2 to 9 dpi, Y(NA) values showed an ascending trends and then were stabilized for all the conditions studied except for the condition of B25-treated barley infected by *D. teres* in which the values remained stable from 2 to 9 dpi. However, this difference in results was not significant (Supplemental Table S1). During a pathogen attack, a lower Y(NA) indicates that PSI is well protected against photoinhibition^57^. Finally, the trends of the results showed that the barley leaf was well protected with strain B25 despite the presence of *D. teres*.

PSI activity is also dependent on the donor side limitation Y(ND)^58^. For all experimental conditions, Y(ND) tended to increase at 2 dpi with significantly different values by comparison two by two with the other times-points (Student’s test; *p* value < 0.05)(Fig. 3b). This increase could be explained by the spraying step. Indeed, the spraying of the pathogen for the conditions with *D. teres* and the spraying of sterile water on the barley plants for the other two conditions can induce an increase of Y(ND) at 2 dpi. The Y(ND) data between the control and the plants infected only with *D. teres* or the B25-bacterized barley were significantly different (Student’s test; *p* value < 0.05) (Supplemental Table S1).

Y(I) tended to decrease from − 4 to 0 dpi then to stabilize its values from 0 to 9 dpi (Fig. 3c). The barley plants bacterized with strain B25 and infected with *D. teres* had the highest values of Y(I) for the different measurement points with significant differences compared to other conditions (Supplemental Table S1). Theoretically, the decrease in Y(ND) and the increase in Y(NA) are caused by photoinhibition of PSI^59^. PSI photoinhibition is an irreversible reaction and is qualified as very costly for photosynthetic plants^60^. Therefore, the low values of Y(NA) obtained thanks to measurements close to the necrotic areas in barley plants bacterized with strain B25 and infected with *D. teres* indicated that PSI is well protected against photoinhibition even in the presence of the pathogen. According to the results obtained, the treatments with *D. teres* or strain B25 allowed to maintain higher values compared to the control plants with a more stable maintenance for the “*D. teres* + strain B25” condition. This suggests that any treatment performed on barley leaves increase the yield of the PSI.

In the leaf, PSI activity is principally dependent on the PSII activity. In addition, PSII is considered to be more vulnerable than PSI in stressed plants^48,61^. Y(II) presented stable values (60–70%) for all the experimental conditions with a slight decrease at 2 dpi with significantly different values by comparison two by two with the other times-points (Student’s test; *p* value < 0.05)(Fig. 3d and Supplemental Table S1). The obtained results showed also that a decrease of Y(II) is correlated with an increase of the Y(ND) parameter in barley leaves.

Y(NPQ) parameter representing the quantum yield of regulated non photochemical energy loss in PSII, tended to increase at 2 dpi for the control condition and infected plants with significantly different values by comparison two by two with the other times-points (Student’s test; *p* value < 0.05) (Fig. 3e). The means of the parameter Y(NPQ) were higher for the controlled plants with significant differences compared to the plants infected by *D. teres* and the bacterized-infected plants (Supplemental Table S1).

The last parameter of PSII, Y(NO) was relatively stable during this experiment (Fig. 3f). Indeed, for all the experimental conditions and for each post-infection times, Y(NO) was around 25–30%. Therefore, the stable Y(NO) values in leaf indicated no increased excitation pressure in PSII reaction centers. According to Klughammer and Schreiber^62^, these results suggest that protective regulatory mechanisms were efficient during biotic stress in barley.

In barley leaf, the PSII photochemistry capacity of the leaf depends particularly on the quantum yield of regulated energy dissipation Y(NPQ), since Y(NO) was stable during our experiment. These higher Y(NPQ) values in non-inoculated plants were different with the obtained values in Fig. 1. In line with the results of this study, the results obtained during the monitoring of photosynthetic performance in wheat after infection with *Pyricularia oryzae* have indicated a decrease in Y(NPQ) compared to non-inoculated plants^63^. Generally, stress induced on the plant decrease the Y(NPQ) parameter. Consequently, the treatments carried out on the barley plants decreased Y(NPQ) compared to the control barley plants which maintained higher values. However, this mechanism is regulated by the dissipation of excessive excitation energy into harmless heat allowing protect plant. Indeed, Y(NPQ) indicated the regulated thermal energy dissipation. Therefore, Y(NPQ) could be stimulated by the xanthophylls cycle if excitation energy is in excess. Without this energy dissipation, singlet O2 and other reactive oxygen species could be formed and therefore, allowed an increase of Y(NO) parameter^58^. In this way, the alterations caused by the pathogen probably reflect the inability of the barley to regulate it mechanisms of photoprotection, resulting in photooxidative damage to the infected host tissue. This regulation was less implemented in the presence of strain B25. Therefore, this result suggested that bacterized-plant implemented less photoprotection mechanisms and did not stimulate the xanthophylls cycle. This result with strain B25 could be explained by the fact that the reaction centers in the thylakoid membrane were open, which led to less activity of NPQ process to compared to the ones closed in control barley^64,65^.

**Figure 4:**
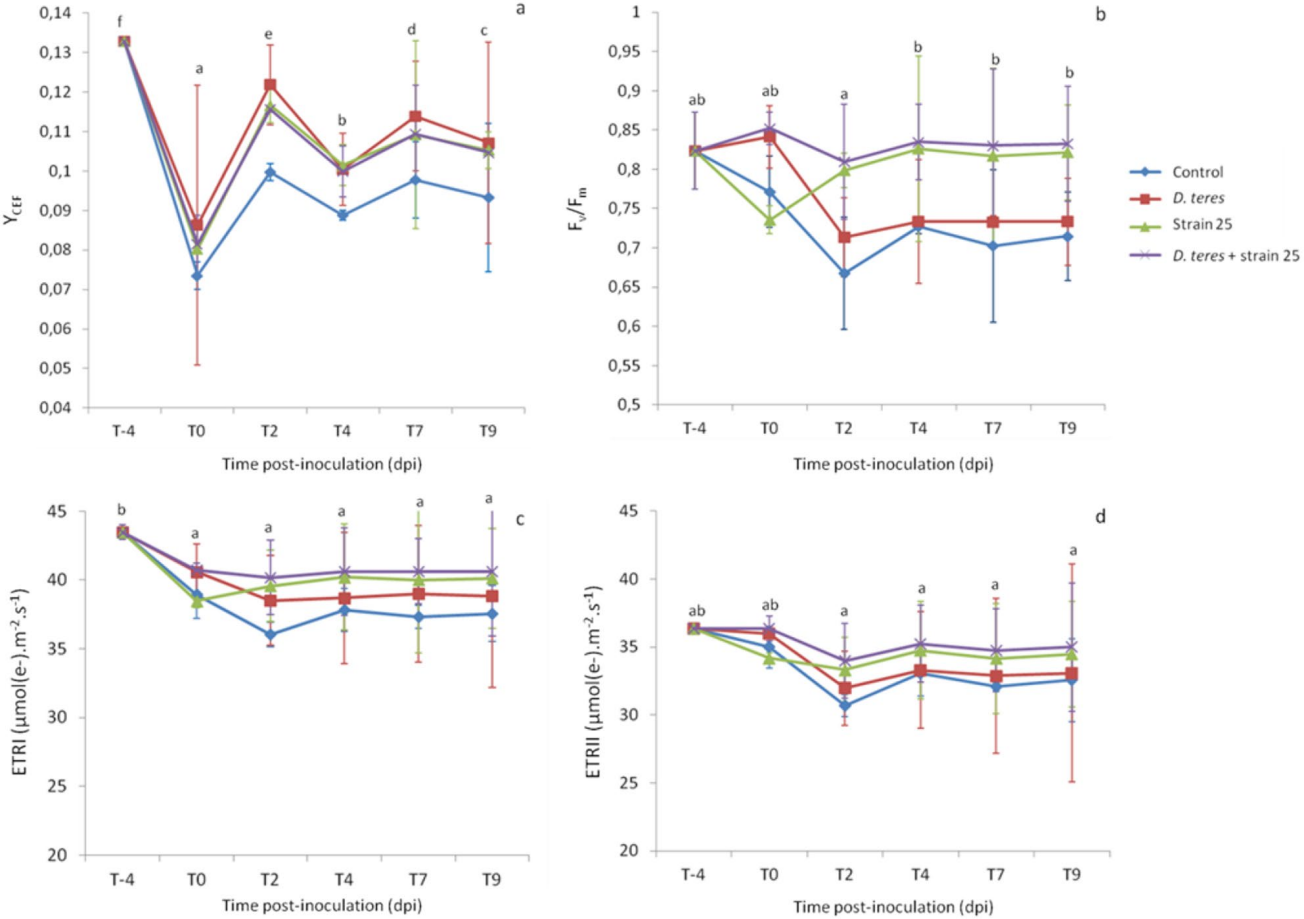
Changes in (**a**) quantum yield of cyclic electron flow (Y_CEF_), (**b**) quantum yield of linear electron flow or maximum efficiency of PSII photochemistry (F_v_/F_m_), (**c**) electron transport rate at PSI reaction centers (ETRI) and (**d**) electron transport rate at PSII reaction centers (ETRII) in the first barley leaf at 4 day before inoculation (T-4) with *D. teres* and 0, 2, 4, 7 and 9 dpi noted T2, T4, T7 and T9, respectively. The mean ± SE was calculated from three independent experiments for each experimental condition and for each time-point, n = 15. Different letters indicate statistically different means (Student’s test; *p* value < 0.05) between times points.

Induction of Y_CEF_ and increase of PSII activity, ETRI and ETRII occurred in the presence of the strain B25. Our monitoring data for the YCEF parameter calculated from Y(I) and Y(II) indicates a decrease for the infection day (T0) with significantly different values by comparison two by two with the other time-points (Student’s test; p value < 0.05). This result can be explained by the spraying step which can induce stress on the barley even without pathogen (Fig. 4a). Then, YCEF of each experimental condition were not relatively stable from 2 to 9 dpi. However, in the presence of *D. teres*, the strain B25 or both of them, YCEF values were significantly increased compared to the control barley leaf from 2 to 9 dpi (Supplemental Table S2). The linear electron transport leads to the generation of ATP as well as NADPH while cyclic electron flow (CEF) allows only the synthesis of ATP mediated by PSI^66^. Under abiotic or biotic stress, the balance between ATP/NADPH production and their consumption may be disturbed and can lead to photodamage and photoinhibition. This balance can be adjusted by upregulation of CEF. For instance, a study demonstrated that heat stress induces an increase of cyclic electron flux around photosystem I in grape leaves^67^. According to these obtained results, the infection and bacterization activate the CEF in comparison to the control to protect the barley during a biotic stress. These results can suggest that the presence of strain B25 increased CEF since the plant has put in place these defense systems identifying the bacterium as a pathogen. In addition, defense systems were put in place when *D. teres* was detected after pulverization with strain B25, allowing the plant to better protect itself after the pathogen attack.

In the photosynthetic reactions, the electrons can follow two pathways. The electrons can be transferred all the way from water to NADPH^+^ accompanied by the production of NADPH and ATP. This first pathway is designated as linear electron flow (LEF). On the contrary, the electrons can follow the cyclic electron flow (CEF). This way recycles electrons around PSI by re-routing them from ferredoxin (Fd) to the plastoquinone (PQ)^68^. Studies have shown the important role of CEF in the protection of PSI in plants under stress. For instance, CEF values increase during treatment of spinach leaves with low temperatures^69^. This result is comparable to that obtained in our study because infection by *D. teres* increased the Y_CEF_ values compared to other experimental conditions.

Concerning the maximum efficiency of PSII photochemistry (F_v_/F_m_), the values of this parameter tended to decrease to 2 dpi and only in the control and plants infected by *D. teres* with significantly different values with 4, 7 and 9 dpi (Fig. 4b). From 2 to 9 dpi, this parameter showed stability with higher values in the presence of the bacterial strain B25 (0.83) compared to the other conditions (0.73). F_v_/F_m_ displayed consistent results with Fig. 1 for measurements taken close to the area of necrosis, as well as with Fig. 2. Indeed, F_v_/F_m_ corresponding to the measurements in the night condition in Fig. 2 was similar with lower values for the control conditions. For F_v_/F_m_ parameter, the treatment with *D. teres* on barley leaves tended to decrease from 0 to 2 dpi according to data obtained close to the necrosis area (Fig. 4b) and was similar to the obtained results according to the Fig. 1.

Electron transport rate at PSI (ETRI) decreased from − 4 dpi to 0 dpi with lower ETRI means for the control condition compared to the other experimental conditions (Fig. 4c; Supplemental Table S2). In addition, ETRII was relatively stable throughout the experiment (Fig. 4d). ETRII means were not significantly different between the experimental conditions (Supplemental Table S2).

According to Su et al*.*^48^, *Pseudomonas syringae* pv. *tomato* decreased F_v_/F_m_, ETRI and ETRII in *Arabidopsis*. In this study, an inoculation of *P. syringae* pv. *tomato* in combination with *Burkholderia phytofirmans* PsJN did not display any variation in photosynthetic activity compared to plants inoculated with the pathogen alone^48^. These results are opposites compared to ours since the bacterial strain B25 increased ETRI, ETRII and F_v_/F_m_. Therefore, some bacterial strains used as biological control agents for plant diseases show improvements in the photosynthetic capacity of the plant.

### Evolution of leaf gas exchange parameters in the presence of the pathogen and the beneficial bacterium strain B25

In general, the symptoms caused by the pathogen on the leaf, decrease gas exchange parameters values because the defenses are set up^25,36,43,67–70^. All the data described below show the effects of the pathogen and the strain B25 on the photosynthetic parameters of barley close to the areas of necrosis.

**Figure 5:**
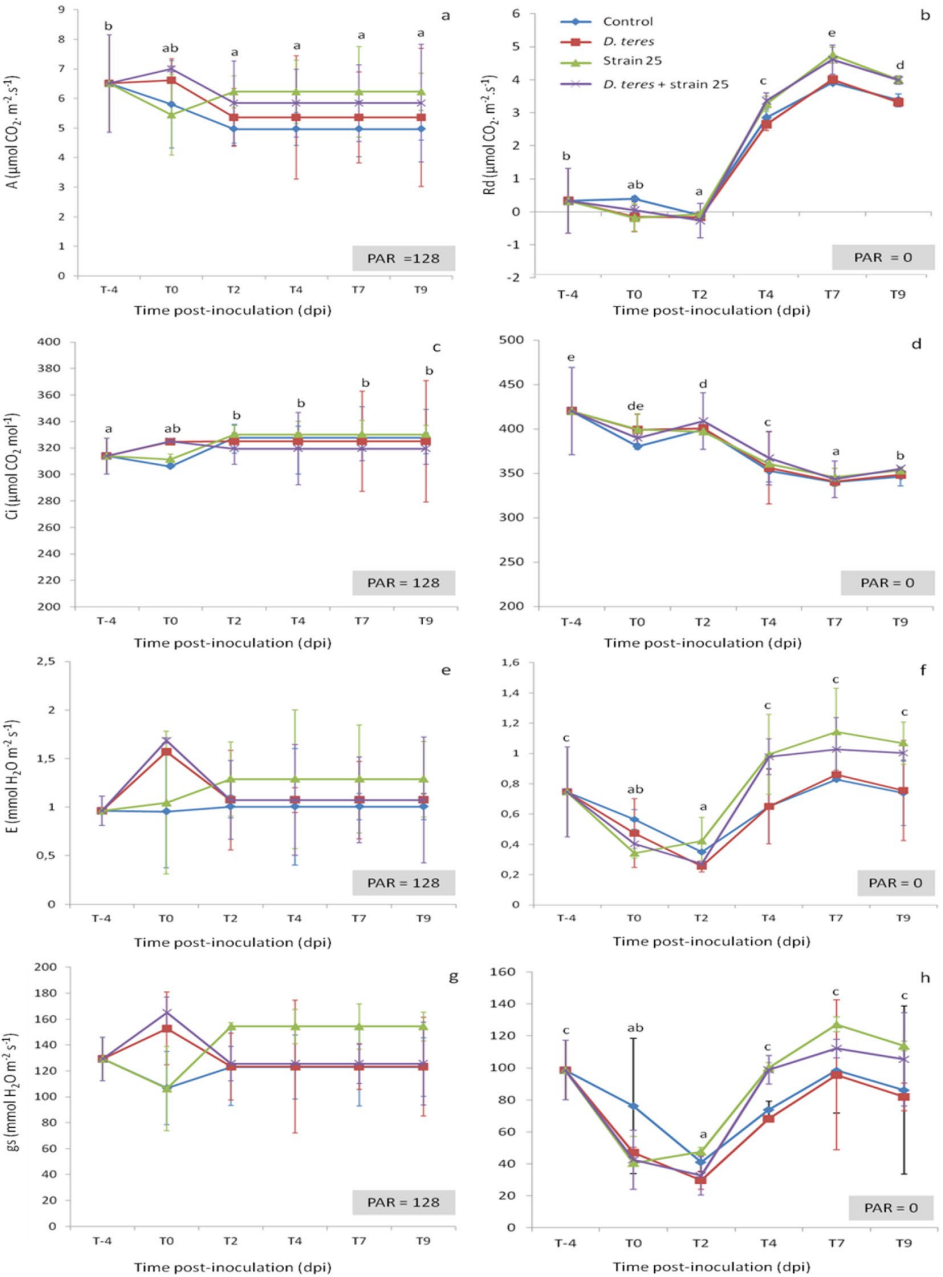
Changes in net carbon assimilation rate (*A*) in day condition (**a**), dark respiration (*Rd*) (**b**), internal CO_2_ concentration (*C*i) in day condition (**c**) and in night condition (**d**), transpiration rate (*E*) in day condition (**e**) and in night condition (**f**) and stomatal conductance (*g*s) in day condition (**g**) and in night condition (**h**) determined on the leaflets of barley plants non-inoculated (control) or inoculated (*D. teres*) or bacterized with strain B25 or inoculated and bacterized (*D. teres* + B25). The measurements were carried out in the first barley leaf 4 days before inoculation (T-4) with *D. teres* and 0, 2, 4, 7 and 9 dpi noted T2, T4, T7 and T9, respectively. Bars represent the standard error of the means, calculated from three independent experiments for each experimental condition and for each time-point, n = 15. Different letters indicate statistically different means (Student’s test; *p* value < 0.05) between times points.

According to our results, the net carbon assimilation rate (*A*) decreased after inoculation with *D. teres* (T0) except for barley plants bacterized with strain B25 but these values were not significant (Fig. 5a). From 2 to 9 dpi, *A* remained stable with values between 5 and 6 µmol m^−2^ s^−1^ for all experimental conditions. In the presence of the bacterial strain B25, the *A* parameter means were higher compared to the control barley leaf and to the barley infected with *D. teres* (Fig. 5a). However, these results were not significantly different (Supplemental Table S3). In addition, the *A* values for the control plants were lower compared to other experimental conditions with significantly differences with “*D. teres*” condition and “*D. teres* + strain B25” condition. With a PAR equal to 0, *A* parameter representing the dark respiration (*Rd*) decreased for all experimental conditions from -4 dpi to 2 dpi. From 2 to 7 dpi, the *Rd* values increased exponentially until 4.7 µmol m^−2^ s^−1^, then decreased slightly with values between 3.3 and 4 µmol m^−2^ s^−1^ from 7 to 9 dpi for all experimental conditions (Fig. 5b). Moreover, towards the end of the experiment (4–9 dpi), *Rd* values were higher for B25 condition and for the infected condition in combination with strain B25, but these results were not significantly different (Supplemental Table S3).

For *Ci*, values remained stable (330 µmol CO_2_ mol^−1^) for all experimental conditions with no significantly results between experimental conditions but with significantly different averages at -4 dpi compared to 2, 4, 7 and 9 dpi (Fig. 5c).

With a PAR equal to 0, *Ci* values decreased for all the experimental conditions without significant differences between conditions but with significantly differences between each time-point (Student’s test; *p* value < 0.05) (Fig. 5d and Supplemental Table S3). With a high PAR (128 µmol m^−2^ s^−1^) or zero, *E* and *gs* parameters showed similar profiles. The presence of *D. teres* increased the *E* and *gs* values at 0 dpi but these results were not significantly different between experimental conditions. Then, values decreased, then remained stable from 2 dpi (Fig. 5e–h). However, these results are significantly different by pairwise comparison between 0, 2 dpi and 4, 7, 9 dpi (Student’s test; *p* value < 0.05).

These results are similar to those obtained by Dias et al*.*^28^. Indeed, the infection with *C. truncatum* has little effect on *A* and *g*s parameters compared to non-inoculated plants^28^. In contrast, other interactions observed a decrease crop yield and a reduction of the rate of carbon fixation of infected leaf tissues. For instance, during the interaction between bean and anthracnose, *A* values are strongly reduced with a decrease of more than 50% when the disease severity is 10% and a value close to zero for a severity greater than 20%^71^. According to Alves et al*.*^40^, *A* decreased in *Eucalyptus urophylla* leaves infected by *Puccinia psidii* and this decrease is proportional to the damage observed in the asymptomatic leaf area^40,72^. The presence of *Stenocarpella macrospora* on maize plants leads to a significant decrease of net CO_2_ assimilation rate, stomatal conductance and transpiration ratio, while the internal to ambient CO_2_ concentration ratio increased in inoculated plants compared to non-inoculated ones^73^. The reduction of the *E* parameter is directly related to *g*s and therefore it is associated with stomatal closure in these pathosystems^71^.

Our results indicate that *A* has higher values in the presence of the beneficial bacterium B25. These results are in line with the fact that beneficial microorganisms appear generally to play a protective role in the functioning of the photosynthetic apparatus. For instance, *Glomus etunicatum*, an arbuscular mycorrhizae fungi appears to increase stomatal conductance and improves transpiration^74^. In addition gas exchanges are stimulated on tomato after plant bacterization with strain PsJN^75^. Despite the current attention on the beneficial microorganisms used as a biocontrol agent, few information are available on the effects of beneficial microorganisms inoculation on photosynthetic capacities and gas exchanges^76^.

In general, an assimilation decrease is observed mainly around the lesion caused by the pathogen while the green tissues present on the infected leaf are not significantly affected for attack by pathogens^77^. The phenomenon of "virtual lesions" is described and shows that the infection with pathogens reduces photosynthesis, not only by reducing the green areas, but also by affecting the assimilation rates and leaf gas exchange in the remaining green areas ^77^. Our results confirm this phenomenon since Fig. 1 shows that the necrotic regions no longer show any photosynthetic activity. By measuring close to the areas of necrosis, the phenomenon of virtual lesions was visualized notably for the Y(NO) and Y_CEF_ parameters. Therefore, *D. teres* affects the photosynthetic capacity in the parts showing symptoms as well as the area close to the necrotic tissue.

## Materials and methods

### Antifungal activity of strain B25

Antifungal activity of strain B25 against *D. teres* was demonstrated by culture on PDA plates, and by detached barley leaves assay. Briefly, 1 cm^2^ of agar plug coated on its surface with young mycelium of *D. teres* was transferred to PDA plates. One day later, 5 µL of suspension of strain B25 (DO_600nm_ = 0.8) was deposited on the same PDA plates with *D. teres*. Then, plates were incubated at 30 °C for 6 days. In addition, two “control” plates were produced; one to show the optimal development of *D. teres* on the PDA plate and the other to ensure the sterility of the medium, respectively. Three biological replicates were performed.

For pathogen infection and disease evaluation, detached leaves assay was carried out. Briefly, barley leaves were disinfected using the following protocol: soaking for 30 s in a 70° alcohol bath then rinsing in three successive baths of sterile water. Leaves were then injured with a wooden pick. This step allows showing the effects of the strain B25 by direct contact. After that, a volume of 10 µL of suspensions containing *D. teres* spores at a concentration of 10^5^ spores/mL was deposited at the leaf wound for the infected condition and 10 µL composed of a mixture of *D. teres* spores at a concentration of 10^5^ spores/mL with a suspension of strain B25 at a concentration of 10^8^ CFU for the bacterized and infected condition. For the control condition, a volume of 10 µL of sterile water was deposited at the level of the wound. Three biological replicates were performed and the symptom reading was taken after 5 days of development at room temperature.

### Plant material, growth conditions and treatments

Barley seeds (cultivar Siberia) were sown in plastic pots containing 90 ± 5 g of substrate Gramo Flor. The barley plantlets were grown in incubators (Aralab) with the following controlled conditions: 23 °C for 14 h of light and 22 °C for 10 h in the dark, the entire day being at 80% relative humidity. The PAR measured in the controlled chamber was 130 µmol m^−2^ s^−1^. The bacterial solution of strain B25 was cultivated in LB medium (tryptone 10 g/L; yeast extract 5 g/L; NaCl 5 g/L; pH 7.2) at 30◦C. For plant bacterization experiments, the cultures were collected after centrifugation at 4500*g* for 15 min, washed, and resuspended in phosphate-buffer saline (PBS) (10 mM of NaH_2_PO_4_, 2.7 mM of KCl, 1.8 mM of KH_2_PO_4_, 137 mM of NaCl, pH 6.8. On 7-day-old barley plants, the strain B25 suspension^78^ was sprayed at a concentration of 10^9^ CFU/mL on the leaves. Control plants were treated with sterile PBS. Three days after bacterization, a suspension of *D. teres* spores at a concentration of 4000 spores/mL diluted in sterile water was sprayed on the barley leaves. In total, this present study includes four experimental conditions: control barley, barley infected with *D. teres*, barley bacterized with strain B25 and barley infected with *D. teres* in the presence of the strain B25. Each experimental condition had 16 pots each pot with 10 seeds. Three biological replicates were carried out for all the experiments described below.

### Chl *a* fluorescence imaging

Images and parameters of Chl *a* fluorescence were obtained on the first leaflets of each plant, from base to apex, per replication and of each treatment at 3, 4, 5, 7 and 10 dpi using the Imaging-PAM (MAXI version) and the imaging fluorometer software ImagingWin (Heinz Walz GmbH, Effeltrich, Germany). Three independent biological replicates were performed (n = 10). The Chl *a* fluorescence emission transients were captured by a CCD (charge-coupled device) camera coupled to the fluorescence device. Initially, the barley leaves were dark-adapted for 30 min after which they were carefully and individually fixed in support. The leaf tissue was then exposed to a saturating flash (2 500 µmol m^−2^ s^−1^, 1 s). This flash allows determine the minimal level of fluorescence (F_0_) when all of the PSII reaction center are “open” and ensure the maximum fluorescence emission (F_m_) when all of the PSII reaction centers are “closed”. Next, actinic illumination (100 µmol m^−2^ s^−1^) was applied after fluorescence stabilization. A second saturating flasf (2 s) was imposed to determine the maximal fluorescence (F_m_’) of light-adapted plantlets leaves. Removal of the actinic light and exposure to a short period of far-red allowed measurement of the zero level of fluorescence (F_0_′). From these initial measurements, the maximal PSII quantum yield (F_v_/F_m_) is estimated as follows (Owera et al.^39^; Rolfe and Scholes^25^; Scholes and Rolfe^79^):

1 $${\text{F}}_{{\text{v}}} /{\text{F}}_{{\text{m}}} = \, \left[ {\left( {{\text{F}}_{{\text{m}}} {-}{\text{ F}}_{0} } \right)/{\text{F}}_{{\text{m}}} } \right]$$

According to Kramer et al.^46^, the quantum yield of non-regulated energy dissipation Y(NO) and the quantum yield of regulated non-photochemical energy loss Y(NPQ) are calculated as follows:

2 $${\text{Y}}\left( {{\text{NO}}} \right) \, = \, \left[ {{\text{F}}/{\text{F}}_{{\text{m}}} } \right]$$

3 $${\text{Y}}\left( {{\text{NPQ}}} \right) = \left( {{\text{F}}/{\text{F}}_{{\text{m}}}^{\prime } } \right) - \left( {{\text{F}}/{\text{ F}}_{{\text{m}}} } \right)]$$

### Monitoring of fluorescence parameters

Chlorophyll fluorescence was measured and was recorded with two “*MONITORING-PAM* Multi-Channel Chlorophyll Fluorometer” or *MONI-PAM* (Walz, Effeltrich, Germany). Each measuring system comprises seven emitter-detector units (*MONI-HEAD*/485) and representing an independent fluorometer. RS-485 serial data communication via a storage-capable (1 GByte memory on microSD flash card) data acquisition system (*MONI-DA*) to a *MONI*-IB4/LAN central interface box allows a communication between the *MONI-HEAD*/485 fluorometers^80^. The *MONI-PAM* system uses modulated blue LED light to measure the fluorescence emitted from a leaf sample. More specifically, the *MONI-HEAD* delivers a single blue Power LED to the sample through a window that transmits radiation in the range of 400–750 nm, situated at the end of the cylinder. The same blue LED emits actinic light and saturating flashes as well as measuring light: the LED emission maximum and full width at half maximum is 455 nm and 18 nm, respectively. Measuring pulses to excite modulated fluorescence are given at frequencies of 5–25 Hz and 100–500 Hz for measurements of fluorescence under low PAR level and high PAR level, respectively.

First barley leaves were fixed in the *MONI-HEAD*’s clip consisting of two aluminum frames (35 × 25 mm). The leaves were placed in such a way to measure all parameters in the leaf area located below the necrotic part. All *MONI-HEAD* were set to be the same height to the neon lights in the incubator. In this present study, the intensity of the saturating light pulses was 3500 µmol photons m^−2^ s^−1^, and the duration of the pulse was 1 s. Saturating pulse analysis detected and calculated fluorescence parameters of barley leaves automatically^55^.

The *MONI-PAM* was operated with Win-Control-3 from a computer. For each measuring point, the instrument recorded the quantum yield of photochemical energy conversion in PSII or Y(II), the electron transport rate (ETR) and photosynthetically active radiation (PAR). These three parameters are related to each other according to the following equation:

4 $${\text{ETR}} = {\text{Y}}\left( {{\text{II}}} \right) \times {\text{PAR}} \times 0.{5} \times {\text{abs}}$$

where 0.5 is the fraction of absorbed light reaching PSII and abs is absorbed radiance taken as 0.84 of incidence radiance^47,81^.

The monitoring (day and night) of the Y(II) parameter is carried out thanks to the measurements obtained every 20 min during 9 days with the *MONI-PAM* probes. Three MONI-HEAD measured the condition of control barley or barley bacterized and four MONI-HEAD measured the condition of infected barley or infected barley in the presence of bacteria. This number of measurement heads represents the number of technical replicates for the three biological replicates carried out. The monitoring was done on four experimental conditions: barley alone (control), barley infected with the pathogen (*D. teres*), and barley bacterized with *Burkholderia* sp. strain B25 (B25) and barley infected with *D. teres* and in presence of the strain B25 (*D. teres* + B25).

### Simultaneous measurements of PSI and PSII parameters

Dual PAM-100 and GFS-3000 systems equipped with a 3010-DUAL gas exchange cuvette (Heinz Walz, Effeltrich, Germany) allowed measuring Chl fluorescence absorbance and gas exchange. The measurements were taken 4 days before infection by D. teres (− 4 dpi), the day of infection (0 dpi) and at 2, 4, 7 and 10 after infection (2, 4, 7 and 10 dpi, respectively). In total, this present study includes four experimental conditions: control barley, barley infected with *D. teres*, barley bacterized with strain B25 and barley infected with *D. teres* in the presence of bacterial strain B25. Three independent experiments were performed for each experimental condition and for each time-point, n = 15. The barley leaves were placed at the cuvette to measure the Y(I) and Y(II) parameters below the zone of necrosis. The barley leaves were dark-adapted for 30 min to determine the minimal level of fluorescence (F_0_) and the maximal fluorescence (F_m_) after a saturating flash (1 s; 13 000 µmol m^−2^ s^−1^). The barley leaves were then exposed to an actinic illumination of 216 µmol m^−2^ s^−1^. A second saturating flash was imposed to calculate the maximal fluorescence (F_m_′) of light-adapted leaves after fluorescence stabilization. The measurement of the zero level of fluorescence (F_0_′) was determined by the removal of the actinic light following by the exposure to a short period of far-red light. According to Genty et al*.*^82^ and Schreiber et al*.*^83^, the fluorescence parameters were calculated in both dark- and light-adapted states.

The quantum yield for photochemical energy utilization in PSII (Y(II)), the quantum yield for regulated energy dissipation in PSII (Y(NPQ)) and the quantum yield for non-regulated energy dissipation in PSII (Y(NO)) have been estimated according to Kramer et al*.*^46^ (Eqs. 1, 2, 3). Y(II) was calculated according to the equation of Genty et al*.*^82^ and note that^84^:

5 $${\text{Y}}\left( {{\text{II}}} \right) \, + {\text{ Y}}\left( {{\text{NPQ}}} \right) \, + {\text{ Y}}\left( {{\text{NO}}} \right) \, = { 1}$$

The electron flow through PSII (ETRII) and the ratio of variable to maximal fluorescence (F_v_/F_m_) are also calculated thanks to Dual PAM-100 and GFS-3000.

Besides measuring the P680 parameters, the saturation pulse method has determined the P700 parameters. The maximum level P_m_ represented P700 fully oxidized and has been determined by application of a saturation pulse after far-red pre-illumination. After the saturating flash and the stop of the far-red illumination, the complete reduction of P700 is induced and represented the minimal level of P700 absorption, P_0_. An intermediate level of absorption is characterized by a fraction of reaction centers that is oxidized in the presence of actinic light. In addition, the parameter P_m_’ corresponds to the absorption level induced by a saturating flash in the presence of actinic light^84^.

Excitation energy reaching PSI centers can either lead to photochemical charge separation with quantum yield Y(I) or be non-photochemically converted to heat. Indeed, P700 is either oxidized (donor side limitation) or no charge separation is possible due to acceptor side limitation^84^. The non-photochemical quantum yield of PSI due to donor-side limitation and designated by Y(ND) is calculated as:

6 $${\text{Y}}\left( {{\text{ND}}} \right) = { 1} - {\text{ P7}}00_{{{\text{red}}}}$$

The quantum yield of non-photochemical energy dissipation due to acceptor-side limitation designated by Y(NA) is determined according to :

7 $${\text{Y}}\left( {{\text{NA}}} \right) = {\mkern 1mu} \left[ {\left( {{\text{P}}_{{\text{m}}} - {\text{P}}_{{\text{m}}} ^{\prime } } \right)/{\text{P}}_{{\text{m}}} } \right]$$

From Y(NA) and Y(ND), Y(I) representing the photochemical quantum yield of PSI is calculated according to this equation:

8 $${\text{Y}}\left( {\text{I}} \right) = { 1} - {\text{ Y}}\left( {{\text{ND}}} \right) \, {-}{\text{ Y}}\left( {{\text{NA}}} \right)$$

The electron flow through PSI (ETRI) is also calculated thanks to Dual PAM-100 software. In addition, the yield of cyclic electron flow (Y_CEF_) is determined as follows^85^:

9 $${\text{Y}}_{{{\text{CEF}}}} = {\text{ Y}}\left( {\text{I}} \right) \, {-}{\text{ Y}}\left( {{\text{II}}} \right)$$

### Determination of barley leaf gas exchange

Simultaneously to PSI and PSII measurements, gas exchanges were measured by Dual PAM-100 and GFS-3000 systems using equations developed by von Caemmerer and Farquhar^86^. At − 4, 0, 2, 4, 7 and 9 dpi, each treatment was measured with a total of five different barley leaves for each experimental condition and for each time-point. In total, this present study includes also four experimental conditions, the same as described above. Three independent experiments were performed allowing obtaining the average of 15 plants for each experimental condition, for each time-point. The same plants and the same measurement area (close to the necrosis area) were used for all measurements and have been dark-adapted during 30 min. Photochemically active radiation provided by a red-blue light emitting diode is fixed at 216 µmol (photons) m^−2^ s^−1^. The absolute pCO_2_ and pO_2_ were maintained at 400 ppm, and 17,000 ppm, respectively, and the cuvette temperature was maintained at 20 ± 1 °C. Net CO_2_ assimilation rates (*A*), stomatal water vapor conductance (*g*s), transpiration rate (*E*), intercellular CO_2_ concentration (*C*i) and the dark respiration (*Rd*) without light over 1.3 cm^2^ rectangular leaf area were measured according to manufacturer specifications. Air flow through the cuvette was exactly adjusted at 400 µmol s^−1^ for all samples. The relative humidity of the gas entering the leaf chamber was set at 70%.

### Statistical analysis

All experiments are repeated independently three times and the standard error of the means is shown. Statistical analyses were performed (*p* value < 0.05) using a two-way analysis of variance (*ANOVA*) to compare the effects of different applied treatments on barley leaves and time responses along the experiment. Student’s tests (*p* value < 0.05) were performed to analyze the results obtained for the several photosynthesis and gas exchange parameters between two tested experimental conditions and two time-points.

### Ethics approval

Experiments involving plants were carried out in compliance with the IUCN policy statement on research involving species at risk of extinction and the convention on the trade in endangered species of wild fauna and flora.

## Conclusion

The measurements of gas-exchange and photosynthetic parameters represent important tools for a better comprehension of the host–pathogen relationships. The results indicate that infection by *D. teres* decreased the maximal photosystem II quantum yield (F_v_/F_m_) and increased the Y_CEF_ parameters representing yield of cyclic electron flow. In addition, the presence of strain B25 allowed maintaining the F_v_/F_m_ parameter. Our study paves the way to follow-up studies of photosynthetic parameters in barley infected by *D. teres* in combination with a biological control agent. The interaction between *D. teres* and strain B25 showed an effect on PSI by acting mainly on Y(NA) and thus allowed better protection of barley against photoinhibition. The infection and bacterization activated the cyclic electron flow. Indeed, the stress and damages induced by *D. teres* increases the cyclic electron flow. Likewise, the presence of the bacterium with or without pathogen also increases this parameter which induces defense mechanisms in barley and thus the host plant can better protect itself against future pathogen attacks. The data presented in this study show the gas exchange variation induced by the several treatments in leaf areas close to the necrosis with an increase the net carbon assimilation rate in presence of the beneficial bacterium B25.

## Supplementary Information

Supplementary Information 1.

## Abbreviations

*A*: Net CO_2_ assimilation rates
CEF: Cyclic electron flow
Chl: Chlorophyll
*C*i: Intercellular CO_2_ concentration
dpi: Days post-infection
*E*: Transpiration rate
ETR: Electron transport rate
*g*s: Stomatal water vapor conductance
F_0_: Minimal chlorophyll fluorescence yield of the dark-acclimated leaves
F_0_′: Minimal chlorophyll fluorescence yield of the light-acclimated leaves
F_m_: Maximal chlorophyll fluorescence yield of the dark-acclimated leaves
F_m_′: Maximal chlorophyll fluorescence yield of the light-acclimated leaves
F_v_: Variable chlorophylle fluorescence
F_v_/F_m_: Maximum quantum yield of PSII photochemistry
NPQ: Non-photochemical quenching of chlorophyll fluorescence
PAM: Pulse-amplitude-modulation
PAR: Photosynthetically active radiation
PBS: Phosphate-buffered saline
PGPR: Plant growth promoting rhizobacteria
P_m_: Maximum oxidation level of P700 obtained from a suturing pulse under far-red light
P_m_′: Maximum amount of photo-oxidized P700 by a saturation pulse at a steady state
PSI: Photosystem I
PSII: Photosystem II
Rd: Dark respiration
ROS: Reactive oxygen species
Y: Quantum yield calculated online with the help of PAM fluorescence
Y_CEF_: Quantum of cyclic electron flow
Y(I): Quantum yield of photochemical energy conversion in PSI
Y(II): Quantum yield of photochemical energy conversion in PSII
Y(NA): Acceptor side limitation
Y(ND): Donor side limitation
Y(NO): Quantum yield of non-regulated non-photochemical energy loss in PSII
Y(NPQ): Quantum yield of regulated non-photochemical energy loss in PSII
ФPS (II): Effective quantum yield of PSII photochemistry
