# Supplementary material for: A biological agent modulates the physiology of barley infected with Drechslera teres
Source: Sci Rep. 2021 Apr 15;11:8330. doi: 10.1038/s41598-021-87853-0 (PMC8050242; doi:10.1038/s41598-021-87853-0)
Supplement: Supplementary file 1 — Supplementary Information 1. [file 41598_2021_87853_MOESM1_ESM.pdf]

Supplemental data

A biological agent modulates the physiology of barley infected with *Drechslera teres*

Aurélie Backes [a], Nathalie Vaillant-Gaveau [a], Qassim Esmaeel [a], Essaid Ait Barka [a], Cédric Jacquard [a,*]

[a] Université de Reims Champagne-Ardenne, Unité de Recherche Résistance Induite et Bio-protection des Plantes, RIBP - EA 4707 - USC INRAE 1488, Moulin de la Housse – Bâtiment 18, BP 1039, 51687 Reims Cedex 2, France ; aurelie.backes@univ-reims.fr (ORCID 0000-0002-7194-2930) (A.B.); nathalie.gaveau@univ-reims.fr (ORCID 0000-0001-5495-7498) (N.V.G.); qassim.esmaeel@univ-reims.fr (ORCID 0000-0002-8085-0800) (Q.E.); ea.barka@univ-reims.fr (ORCID 0000-0002-5138-306X) (E.A.B.)

* Correspondence: cedric.jacquard@univ-reims.fr (ORCID 0000-0003-4284-6082) (C.J.)

Figure S1. Results of the antagonism test on PDA plates showing the antifungal activity of the strain B25 (indicated by a red arrow) against *D. teres*. Negative control without *D. teres* (a), positive control with *D. teres* (b) and the antagonism test on PDA plate with the co-culture of *D. teres* and strain B25 (c). The table indicates the measurements of the inhibition zone of the fungus growth. The inhibition zone corresponds to the radius from the center of the fungus origin spot until the end of mycelium growth caused by the presence of the bacterium. Values of inhibition zone (cm) ± SD represent means of three independent experiments.

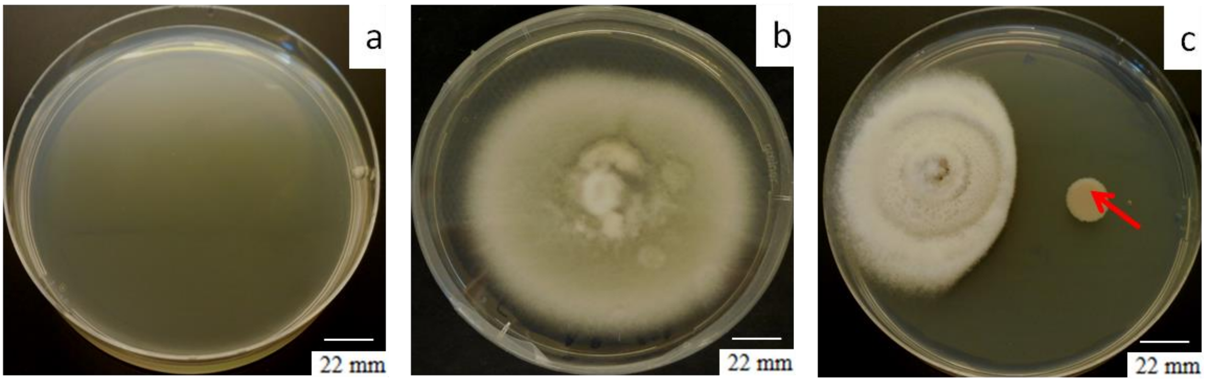

**Biological replicates**	***D. teres* growth (cm)**	**Inhibition zone (cm)**
n° 1	3.25 ± 0.25	1.00 ± 0.15
n° 2	3.65 ± 0.15	1.20 ± 0.15
n° 3	3.65 ± 0.15	0.90 ± 0.15

Figure S2. Results of detached barley leaves assay showing the antifungal activity of the strain B25 against *D. teres*. Negative control without *D. teres* (a), positive control with *D. teres* (b) and the antagonism test on barley leaves with the co-culture of *D. teres* and strain B25 (c). Values of necrosis size (cm) ± SD represent means of three independent experiments.

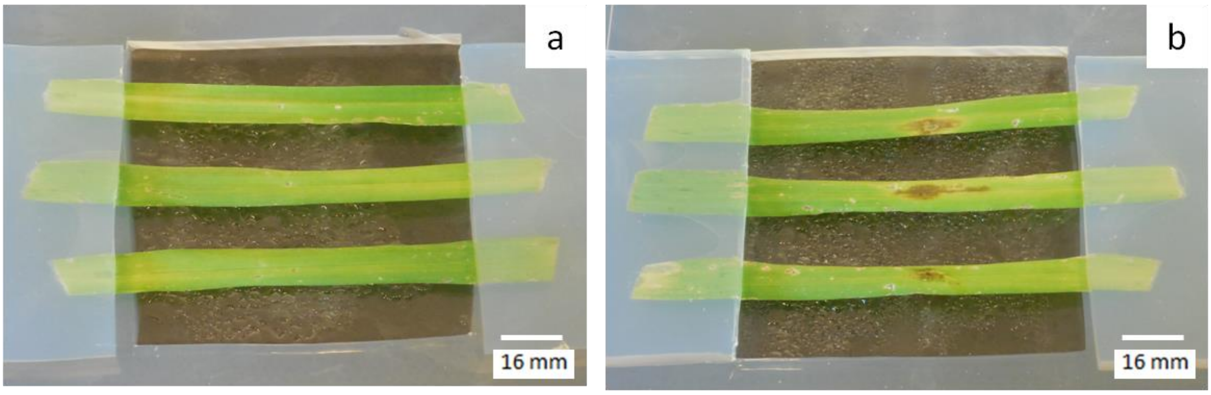

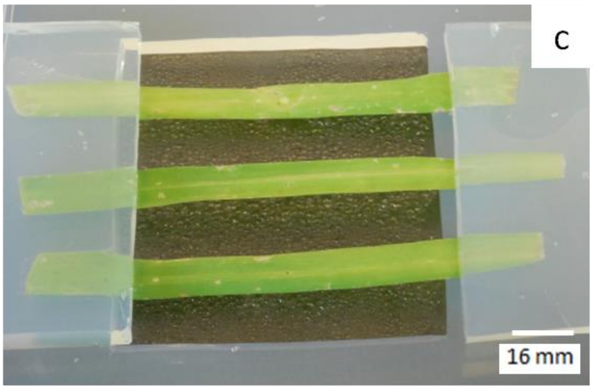

Biological replicates	Infected Control	B25 condition
	necrosis size (cm)	
n° 1	0.73 ± 0.22	0.06 ± 0.08
n° 2	0.80 ± 0.13	0
n° 3	1.26 ± 0.60	0.03 ± 0.03

Table S1. Results of a two-way ANOVA and results of a Student's test of PSI and PSII photosynthetic parameters with significant results at *p*-value < 0.05 (*) and not significant (NS), respectively. The student's test was used to calculate the significant differences between experimental conditions for the same time-point.

	**Parameters**	**Y(NA)**	**Y(ND)**	**Y(I)**	**Y(II)**	**Y(NPQ)**	**Y(NO)**
ANOVA (p-value < 0.05)	Conditions	*	NS	*	NS	*	*
	Time	*	*	*	*	*	NS
Student's test (p-value < 0.05)	Control vs *D. teres*	NS	*	*	NS	*	NS
	Control vs strain B25	NS	*	NS	NS	NS	*
	Control vs *D. teres* + strain B25	NS	NS	*	NS	*	*
	D. teres vs strain B25	NS	NS	NS	NS	NS	NS
	D. teres vs *D. teres* + strain B25	NS	NS	*	NS	NS	*
	Strain B25 vs *D. teres* + strain B25	NS	NS	*	NS	NS	NS

Table S2. Results of a two-way ANOVA and results of a Student's test of Y_{CEF}, F_v/F_m , ETRI and ETRII parameters with significant results at *p*-value < 0.05 (*) and not significant (NS), respectively. The Student's test was used to calculate the significant differences between experimental conditions for the same time-point.

	**Parameters**	**Y_{CEF}**	**F_v/F_m**	**ETRI**	**ETRII**
ANOVA (*p*-value < 0.05)	Conditions	*	*	*	NS
	Time	*	NS	*	*
Student's test (*p*-value < 0.05)	Control vs *D. teres*	*	NS	*	NS
	Control vs strain B25	*	*	*	NS
	Control vs *D. teres* + strain B25	*	*	*	NS
	D. teres vs strain B25	NS	*	NS	NS
	D. teres vs *D. teres* + strain B25	NS	*	NS	NS
	Strain B25 vs *D. teres* + strain B25	NS	NS	NS	NS

Table S3. Results of a two-way ANOVA and results of a Student's test of *A* in day condition, *Rd*, *C*i in day condition and in night condition, *E* in day condition and in night condition and *g*s in day condition and in night condition with significant results at p-value < 0.05 (*) and not significant (NS), respectively. This test allows to identify significantly different results between the experimental conditions for the same time-point.

	Parameters	**A**	**Rd**	**Ci (PAR=128)**	**Ci (PAR=0)**	**E (PAR=128)**	**E (PAR=0)**	**gs (PAR=128)**	**gs (PAR=0)**
ANOVA (p-value < 0.05)	Conditions	*	NS	NS	NS	NS	NS	NS	NS
	Time	*	*	*	*	NS	*	NS	*
Student's test (p-value < 0.05)	Control vs *D. teres*	*	NS	NS	NS	NS	NS	NS	NS
	Control vs strain B25	NS	NS	NS	NS	NS	NS	NS	NS
	Control vs *D. teres* + strain B25	*	NS	NS	NS	NS	NS	NS	NS
	D. teres vs strain B25	NS	NS	NS	NS	NS	NS	NS	NS
	D. teres vs *D. teres* + strain B25	*	NS	NS	NS	NS	NS	NS	NS
	Strain 25 vs *D. teres* + strain B25	NS	NS	NS	NS	NS	NS	NS	NS
